# Addressing conceptual and design gaps in the oncology nutrition evidence base during chemotherapy: contributions of the Exercise and Nutrition Interventions to Improve Cancer Treatment-Related Outcomes Consortium

**DOI:** 10.1093/jnci/djaf143

**Published:** 2025-07-18

**Authors:** Stephanie L E Compton, Heather Wopat, Melissa Lopez-Pentecost, Tanya Agurs-Collins, Justin C Brown, Bette Caan, Wendy Demark-Wahnefried, Joanne W Elena, Leah M Ferrucci, Courtney McGowan, Leah S Puklin, Kathryn H Schmitz, Cynthia A Thomson, Kim Robien, Tracy E Crane, ENICTO Consortium, Bette Caan, Sydney Anderson, Harmenjit Bahia, Adrienne Castillo, Elizabeth Feliciano, Kristina Johnson, Michelle Ross, Erin Weltzein, Justin C Brown, Stephanie Compton, Tamara Green, Phillip Nauta, Shengping Yang, Jeffrey A Meyerhardt, Christina M Dieli-Conwright, Danny Nguyen, Amalia Perez Pena, Guillaume Spielmann, Youyoung Kim, William J Evans, Jennifer W Bea, Robert M Blew, Cynthia A Thomson, Tracy E Crane, Victoria Camacho, Valery Cespedes, Stefan Spee Erlandsen, Sarah Grey Freylersythe, Kenna Hollander, Melissa Lopez-Pentecost, Frank J Penedo, LaShae Rolle, Paola Rossi, Matthew Schlumbrecht, Madalyn Wheeler, Melinda L Irwin, Anlan Cao, Brenda Cartmel, Leah M Ferrucci, Linda Gottlieb, Maura Harrigan, Fang-Yong Li, Courtney McGowan, Leah Puklin, Ratner Elena, Tara Sanft, Michelle Zupa, Nathan A Berger, Stephen Cerne, Carissa Mills, Sandy Conochan, Jasmin Hundal, Cynthia Owusu, John Pink, Jennifer A Ligibel, Nancy Campbell, Kaedryn DiGuglielmo, Wendy Kemp, Christopher Maples-Campbell, Truong Nguyen, Jay Oppenheim, Anna Tanasijevic, Cynthia Thomson, Angela Yung, Karen Basen-Engquist, Preena Loomba, Vernon M Chinchilli, Kathryn H Schmitz, Jenna Binder, Shawna E Doerksen, Julia Foldi, Sara Garrett, Raymond Scalise, Michele Sobolewski, Jessica M Scott, Andrea Cercek, Sheng F Cai, Stephanie Cao, Helena Furberg, Jenna Harrison, Lee W Jones, Catherine Lee, Ross Levine, Meghan Michalski, Chaya S Moskowitz, Amanda O’ Meara, Julia Rabazzi, Kurtis Stoeckel, Talya Salz, Martin R Weiser, Anthony F Yu, Wendy Demark-Wahnefried, Kim Robien, Scott R Evans, Loretta DiPietro, Bao Duong, Sharon L Edelstein, Lorens Helmchen, Daisy Le, Caitlin McCleary, Ashley H Tjaden, Heather Wopat, Borsika A Rabin, Frank M Perna, Tanya Agurs-Collins, Susan M Czajkowski, Joanne Elena, Linda C Nebeling, Wynne E Norton

**Affiliations:** Pennington Biomedical Research Center, Baton Rouge, LA, United States; Milken Institute School of Public Health, George Washington University, Washington, DC, United States; Sylvester Comprehensive Cancer Center, University of Miami, Miami, FL, United States; Division of Cancer Control and Population Sciences, National Cancer Institute, Bethesda, MD, United States; Pennington Biomedical Research Center, Baton Rouge, LA, United States; Lousiana State University Health Sciences Center, New Orleans School of Medicine, New Orleans, LA, United States; Stanley S. Scott Cancer Center, Louisiana State University Health Sciences Center, New Orleans, LA, United States; Division of Research, Kaiser Permanente of Northern California, Oakland, CA, United States; Department of Nutrition Sciences, University of Alabama at Birmingham, Birmingham, AL, United States; Division of Cancer Control and Population Sciences, National Cancer Institute, Bethesda, MD, United States; Yale Cancer Center, Yale University, New Haven, CT, United States; Yale School of Public Health, Yale University, New Haven, CT, United States; Yale School of Public Health, Yale University, New Haven, CT, United States; Yale School of Public Health, Yale University, New Haven, CT, United States; Hillman Cancer Center, University of Pittsburgh, Pittsburgh, PA, United States; Mel and Enid Zuckerman College of Public Health, University of Arizona, Tucson, AZ, United States; Milken Institute School of Public Health, George Washington University, Washington, DC, United States; Sylvester Comprehensive Cancer Center, University of Miami, Miami, FL, United States

## Abstract

Evidence to support the development of practice guidelines on nutrition interventions during active cancer treatment is limited despite the established role of nutrition in cancer prevention and long-term survivorship. To address this gap, the National Cancer Institute funded the Exercise and Nutrition Interventions to Improve Cancer Treatment-Related Outcomes (ENICTO) research consortium. This manuscript focuses on the nutrition-specific work within the ENICTO Consortium. We present a conceptual framework describing how nutritional interventions may enhance cancer treatment tolerance and timely completion of chemotherapy. We also describe how each ENICTO research project selected specific nutrition-related data items and collection methods to test hypotheses outlined in the conceptual framework. Research and consortium-wide projects are described in relation to advancing the scientific rigor of research in the field, including the standardization of nutrition assessment tools and measures. We conclude with a call to action for further research to support the development of evidence-based oncology nutrition practice guidelines relevant to the treatment period within the cancer continuum.

## Introduction

Despite a large body of evidence supporting the role of nutrition in cancer prevention and long-term survivorship, evidence-based guidance for nutrition during active cancer treatment is limited.^1^ Interventions during active treatment present unique challenges beyond those evident in the prevention or long-term survivorship settings. For example, the heterogeneity of cancer types and treatment regimens, each with their own nutrition-related symptoms and dietary needs, adds to the complexity of developing effective nutrition approaches that will ultimately improve treatment response and survival.

Expert reviews by the American Society of Clinical Oncology and the American Cancer Society conclude there is insufficient evidence to make specific nutrition recommendations to prevent and manage malnutrition, reduce treatment-related toxicity, or optimize treatment efficacy during cancer treatment.^2^^,^^3^ In 2022, a total of 5 institutes and offices of the National Institutes of Health cosponsored the Pathways to Prevention (P2P) workshop, entitled Nutrition as Prevention for Improved Cancer Outcomes, to address these research gaps.^1^ As part of the P2P workshop process, the Agency for Healthcare and Research Quality Evidence-Based Practice Center at the University of Minnesota^4^ conducted a systematic review of the scientific literature, framed around 6 key questions. Two of these questions highlighted the need to evaluate the effects of nutrition interventions on preventing negative treatment outcomes and reducing treatment toxicities in patients being treated for cancer.^1^^,^^4^ The review panel emphasized the need for (1) developing conceptual frameworks describing the mechanisms by which nutrition interventions may improve cancer health outcomes; (2) improving rigor in the primary intent, design, and reporting of nutrition studies; and (3) standardizing definitions and taxonomies in nutrition studies for populations, interventions, and outcomes related to nutrition.

This manuscript outlines efforts across a National Cancer Institute (NCI)–funded research consortium designed to address the lack of rigorous research in oncology nutrition: the Exercise and Nutrition Interventions to Improve Cancer Treatment-Related Outcomes (ENICTO) consortium. First, we present the conceptual framework that guided development of nutrition-specific research across the consortium. Second, we address our approach to developing rigorous intent, design, and outcomes reporting for selected cross-consortium nutrition research topics of interest. Third, we describe collaborative efforts to select validated nutrition screening and assessment tools with standard nutrition taxonomy and definitions employed across ENICTO trials. Finally, we discuss the advantages and challenges resulting from these efforts and call for future studies to optimize the potential for nutrition research during active treatment by using validated dietary instruments with standard definitions.

### The ENICTO Consortium

The ENICTO Consortium is an effort by the NCI (RFA-CA-21-032, https://enicto.bsc.gwu.edu/) to address existing knowledge gaps in exercise and nutrition interventions during cancer treatment. The 4 clinical trials of the ENICTO Consortium include participants who have been diagnosed with colon (Adaptive Randomization of Aerobic Exercise During Chemotherapy in Colon Cancer [ACTION], NCT05773144), ovarian (Trial of Exercise And Lifestyle [TEAL], NCT05761561), breast (TeleHealth Resistance exercise Intervention to preserve dose intensity and Vitality in Elder breast cancer patients [THRIVE-65], NCT05535192), and gastrointestinal (Tele-exercise during Neoadjuvant Chemotherapy Trial [TNT], NCT05789485) cancer and have been described elsewhere^5^ (Table S1). A coordinating center supports the consortium through data harmonization and analysis of cross-consortium research questions. The primary research question for each of the ENICTO trials is to determine whether exercise prescriptions alone (TNT and ACTION) or in combination with medical nutrition interventions (THRIVE-65 and TEAL) during chemotherapy impact relative dose intensity. Relative dose intensity is calculated as the ratio of the delivered dose intensity divided by the planned dose intensity times 100 and integrates dose reductions, delays, and early discontinuation.^6^^,^^7^ Additional trial design and outcomes has been described previously.^5^

Unlike other lifestyle intervention consortia in which a common intervention protocol is developed after the participating performance sites are selected, applicants to the ENICTO request for applications proposed independent studies with the common goal of improving cancer treatment tolerance using interventions of exercise alone or in combination with dietary or nutrition-related regimens. The funded projects predominantly focus on exercise interventions, with 2 studies additionally implementing a nutrition-related intervention to improve overall dietary quality (TEAL) or nutrition counseling on adequate protein intake and protein supplementation as needed (THRIVE-65). However, all 4 projects collect data on dietary intake and nutritional status.

#### Diet and Malnutrition Working Group Formation

Following the launch of the consortium in late spring 2022, working groups were convened around key areas of consortium-wide data collection. These working groups focused on identifying domains, including nutrition, where common data elements could be collected across the 4 studies thus optimizing data harmonization and fostering cross-consortium collaborations.

Nutrition researchers from each of the 4 trials and the coordinating center formed the Diet and Malnutrition Working Group (DMWG). Their objective was to prioritize cross-consortium oncology nutrition research topic areas, develop a conceptual framework of mechanisms by which nutrition may alter chemotherapy outcomes, and identify the appropriate data collection and assessment tools needed to evaluate the conceptual framework.

The prioritization of research topics was determined by the existing state of the literature on the topic and feasibility of collecting adequate data within the consortium. The DMWG agreed on 5 main cross-consortium research topic areas, including characterizing the following:

- Dietary intake prediagnosis and/or preintervention and throughout chemotherapy
- Adherence to nutrition interventions and changes in diet quality between baseline and postchemotherapy timepoints
- The prevalence and/or trends in incidence of nutrition impact symptoms (eg, loss of appetite, vomiting) throughout the course of chemotherapy
- The prevalence of malnutrition at baseline and on completion of chemotherapy
- The prevalence of baseline food security status and the association with intervention adherence and relative dose intensity

### Diet and malnutrition conceptual framework

**Figure 1.**
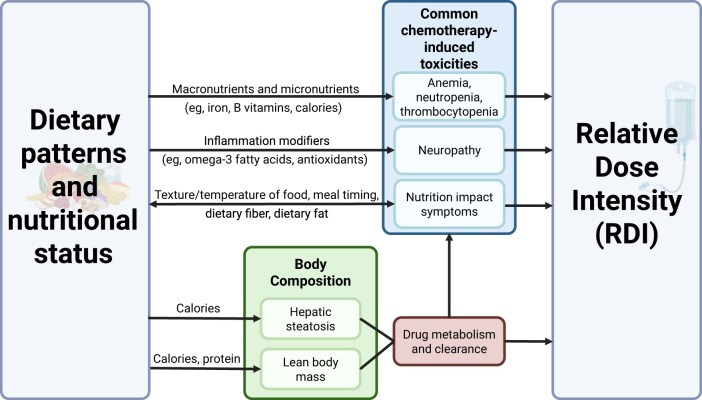
Exercise and Nutrition Interventions to Improve Cancer Treatment-Related Outcomes Oncology Nutrition Conceptual Framework of the relationship between dietary patterns, nutritional status, and chemotherapy relative dose intensity. Created using Biorender.

The DMWG developed a conceptual framework (Figure 1) that outlines hypothesized mechanisms by which dietary patterns and nutritional status may impact chemotherapy relative dose intensity. For example, excessive or inadequate intake of macronutrients (protein, fats, and carbohydrates) may impact relative dose intensity through changes in body composition, which can, in turn, alter pharmacokinetics.^8-10^ Adequate protein and energy intake is necessary to maintain muscle structure and function during cancer treatment,^11^ and adequate calorie and protein intake during treatment may ameliorate or stabilize muscle loss.^12^ Low lean body mass can slow drug metabolism and excretion and is associated with increased chemotherapy toxicity.^9^^,^^10^^,^^13^^,^^14^

Another example of how nutritional status may affect chemotherapy relative dose intensity includes hematological chemotherapy toxicities and neuropathy, which may be impacted by preexisting or treatment-induced deficiencies in micronutrients such as iron,^15^^,^^16^ B vitamins,^17^ and antioxidants.^18^ Anemia in those with cancer can occur because of inadequate dietary iron or vitamin B12 intake, blood loss, or from inefficient iron absorption and metabolism due to inflammation.^16^ Prechemotherapy iron deficiency or low hemoglobin has been found to predict chemotherapy-induced anemia,^19-21^ a type of hematological toxicity that may result in treatment delays, dose or drug changes, or additional medical interventions.^22^^,^^23^

Cancer-related nutrition impact symptoms, such as loss of appetite^24^ or changes in bowel habits,^25^^,^^26^ may occur prior to treatment and increase susceptibility to adverse events of treatment by worsening baseline nutritional status.^27^ Chemotherapy-induced nutrition impact symptoms, including nausea, vomiting, mucositis, and increased sensitivity to odors and metallic taste,^28^ may bidirectionally affect treatment tolerability and nutritional status by altering appetite, food preferences, and tolerance. These nutrition impact symptoms may contribute to preferences for soft, cold foods and aversions to strongly flavored foods such as meat, leafy green vegetables, or heavily spiced food.^28-30^ Patients may find small, frequent meals more tolerable than large meals.^27^ Constipation or diarrhea may prompt patients to respectively increase or decrease soluble fiber and fat intake,^31^ although increasing fiber may worsen early satiety.^27^^,^^32^

The conceptual framework serves as a working model to address relevant nutrition-related topics and emergent research questions in the ENICTO Consortium. Rigorous assessment of dietary quality before and during chemotherapy treatment will provide foundational knowledge to test these proposed mechanisms and tie directly into the key questions raised by the P2P.^1^

### Approach to improving rigor in dietary assessment

#### Selected outcomes and assessment tools

The initial selection of dietary assessment methods for each trial was driven by study design and study-specific factors, including the intervention, individual study objectives, potential participant characteristics, resource constraints, and other logistical considerations. Upon the launch of the consortium, the DMWG facilitated discussion between the trials to collect common nutrition data elements outlined in Table 1. A consortium-wide manual of procedures describes best practices to standardize data collection of the common nutrition data elements across the 4 studies and 8 performance sites.

**Table 1.** Common data elements

Domain	Constructs	ACTION	TEAL	THRIVE-65	TNT	DMWG research topics
Macronutrient intake	Calorie intake, kcal/day	Self-administered ASA24	Interviewer-assisted NDSR 24-hour dietary recall FFQ Food logs	Interviewer-assisted NDSR 24-hour dietary recall Protein checklist	Interviewer-assisted ASA24	Characterizing dietary intake prediagnosis and/or preintervention and throughout chemotherapy and describing adherence to nutrition interventions and maintenance of dietary changes postintervention
	Protein intake, g/day					
	Added sugars, g/day					
	Fiber, g/day					
	Alcohol, g/day					
	Alcohol, drinks/day					
Micronutrient intake	Vitamins, minerals, phytochemicals, and other compounds	Self-administered ASA24	Interviewer-assisted NDSR 24-hour dietary recall Food logs Skin carotenoid level	Interviewer-assisted NDSR 24-hour dietary recall	Interviewer-assisted ASA24	
Food groups	Vegetables, servings/day	Self-administered ASA24	Interviewer-assisted NDSR 24-hour dietary recall FFQ Food logs	Interviewer-assisted NDSR 24-hour dietary recall	Interviewer-assisted ASA24	
	Whole grain, servings/day					
Dietary supplements	Dietary supplements	Self-administered ASA24	FFQ		Interviewer-assisted ASA24	
Temporal eating patterns	First and last eating episode		Interviewer-assisted NDSR 24-hour dietary recall	Interviewer-assisted NDSR 24-hour dietary recall		
Nutrition impact symptoms	Appetite Weight loss Pain Nausea Early satiety Dry mouth Difficulty swallowing Throat and/or mouth sores Taste change Vomiting Heartburn Constipation Diarrhea Abdominal pain Flatulence	PRO-CTCAE	PRO-CTCAE	PRO-CTCAE	PRO-CTCAE	Nutrition impact symptoms throughout the course of chemotherapy
Malnutrition risk	Weight Food intake Symptoms Activities and function	PG-SGA, PRO-CTCAE	PG-SGA, PRO-CTCAE	PRO-CTCAE	PG-SGA, PRO-CTCAE	Malnutrition status
Food security	Within the past 12 months we worried whether our food would run out before we got money to buy more.		Hunger Vital Sign	Hunger Vital Sign	Hunger Vital Sign	Food security
	The food that (I/we) bought just didn’t last, and (I/we) didn’t have money to get more.	USDA-6	Hunger Vital Sign	Hunger Vital Sign	Hunger Vital Sign	
	(I/we) couldn’t afford to eat balanced meals.	USDA-6				
	In the last 12 months, did (you/you or other adults in your household) ever cut the size of your meals or skip meals because there wasn’t enough money for food?	USDA-6				
	How often did this happen—almost every month, some months but not every month, or in only 1 or 2 months?	USDA-6				
	In the last 12 months, did you ever eat less than you felt you should because there wasn’t enough money for food?	USDA-6				
	In the last 12 months, were you ever hungry but didn’t eat because there wasn’t enough money for food?	USDA-6				

Abbreviations: ACTION = Adaptive Randomization of Aerobic Exercise During Chemotherapy in Colon Cancer; ASA24 = Automated Self-Administered 24-hour Dietary Assessment Tool; DMWG = Diet and Malnutrition Working Group; FFQ = Food Frequency Questionnaire; NDSR = Nutrition Data System for Research; PRO-CTCAE = Patient-Reported Outcomes Common Terminology Criteria for Adverse Events; TEAL = Trial of Exercise And Lifestyle; TNT = Tele-exercise during Neoadjuvant chemotherapy Trial; THRIVE-65 = TeleHealth Resistance exercise Intervention to preserve dose intensity and Vitality in Elder breast cancer patients; USDA-6 = US Department of Agriculture Economic Research Service Six-Item Short Form.

**Figure 2.**
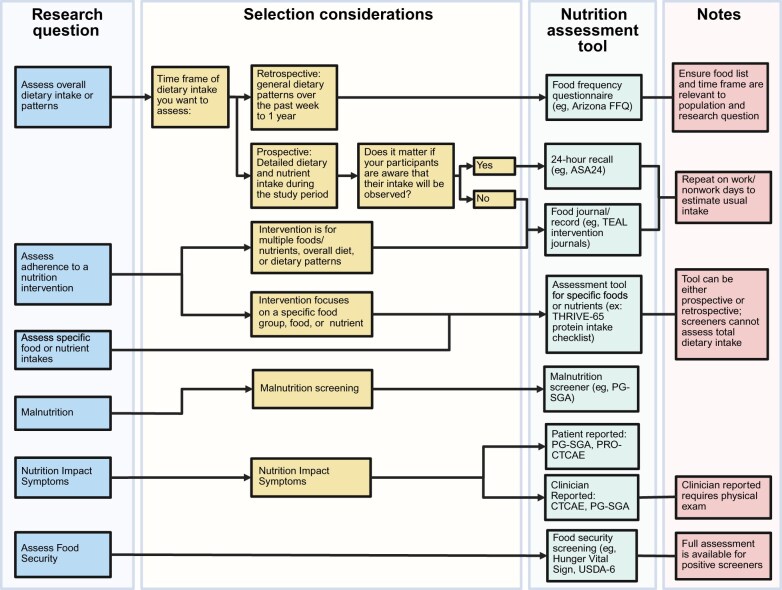
Nutrition assessment tool algorithm. Algorithm for nutrition assessment tools based on research questions and outcomes. Created using Biorender. Abbreviations: ASA24 = Automated Self-Administered 24-hour; FFQ = Food Frequency Questionnaire; PG-SGA = Patient-Generated Subjective Global Assessment; PRO-CTCAE = Patient-Reported Outcomes Common Terminology Criteria for Adverse Events; TEAL = Trial of Exercise And Lifestyle; THRIVE-65 = TeleHealth Resistance exercise Intervention to preserve dose intensity and Vitality in Elder breast cancer patients; USDA-6 = US Department of Agriculture Economic Research Service 6-Item Short Form.

The following sections outline the outcomes of interest based on the identified nutrition-related topics and describe the assessment tools administered by each ENICTO trial. An algorithm created to select nutrition assessment tools based on research questions and selection considerations is presented in Figure 2. The dietary assessment and nutrition impact symptoms instruments that were selected are briefly described in Table 2.

**Table 2.** Instruments for diet screening, dietary intake assessment, and nutrition-impact symptom assessment in ENICTO

Instrument	Parameters	Time frame	Recommended uses and limitations	Domains influencing selection
Screening				
Protein checklist	Frequency of specified protein food intake	Prospective and retrospective, for over 1 week at a time in THRIVE-65	Estimation of protein intake adequacy Limited data collection—cannot assess full dietary patterns just intake of specific foods or nutrients	Low participant burden Low cost Screeners are unable to assess total intake, estimate energy intake, meal timing or setting, or intake of multiple foods and nutrients
Dietary assessment				
FFQ	Foods, beverages, dietary supplements, estimated portion size, frequency	Retrospective, usually evaluates intake for the previous 1, 3, or 6 months. The Arizona FFQ evaluates dietary intake for the previous 6 months in TEAL	Assessment of habitual dietary patterns over a designated period of time Potential for recall or social desirability bias Limited data collection—cannot describe mealtime or setting, specific details of preparation	Can assess long-term retrospective usual dietary intake Cannot precisely estimate energy intake Additional cost per participant Food list must be relevant to the population Inappropriate in populations who are unable to recall the specified time period (children, participants with memory deficits)
ASA24 24-hour recall, with or without interviewer assistance	Foods, beverages, dietary supplements	24 hours	Provides detailed, quantifiable short-term dietary intake and can be used for energy and specific nutrient assessment; can be used to calculate dietary pattern scores	Moderate participant burden: average 24 minutes (17-34) to complete Free or low cost Can be assisted by trained interviewer (recommended) Can estimate short-term retrospective energy and specific nutrient intake
24-hour recall Interviewer-assisted 24-hour dietary recall	Foods, beverages, dietary supplements	24 hours	Provides detailed, quantifiable short-term dietary intake and can be used for energy and specific nutrient assessment; can be used to calculate dietary pattern scores	Moderate participant burden (average 20 minutes, 30 with supplements) Additional cost for assistance by trained interviewer and NDSR database Can estimate short-term retrospective energy and specific nutrient intake Large underlying database
Food log or journal	Foods, beverages, dietary supplements	Daily for specified number of days	Provides detailed, quantifiable short-term dietary intake and can be used for energy and specific nutrient assessment and is not reliant on memory May be used to measure adherence to a dietary intervention	High participant and staff burden May affect dietary habits of participants Can estimate short-term energy and specific nutrient intake
Nutrition impact symptoms				
PRO-CTCAE	Frequency, severity, interference, and presence or absence of symptomatic toxicities (eg, fatigue, nausea, pain)	7 days	Used for precise, reproducible assessment of adverse events and/or nutrition-impact symptoms during cancer treatment	Patient-reported Low or no cost Specific criteria for grading severity Able to include only targeted symptoms to reduce time needed to complete
PG-SGA 4-in-1 instrument for screening, assessment, triage, and monitoring	Patient-reported weight, recent food intake, symptoms, and activities and function with clinician-reported physical exam	2 weeks (nutrition impact symptoms) 1 month (food intake, activities, weight change) 6 months (weight change)	Identify patients who are at risk of or have malnutrition	Low participant burden (4 questions, <5 minutes to complete, easy use) Freely available Requires trained professional for scoring and intervention on findings

Abbreviations: ASA24 = Automated Self-Administered 24-hour; ENICTO = Exercise and Nutrition Interventions to Improve Cancer Treatment-Related Outcomes; FFQ = Food Frequency Questionnaire; NDSR = Nutrition Data System for Research; PG-SGA = Patient-Generated Subjective Global Assessment; PRO-CTCAE = Patient-Reported Outcomes Common Terminology Criteria for Adverse Events; TEAL = Trial of Exercise And Lifestyle; THRIVE-65 = TeleHealth Resistance exercise Intervention to preserve dose intensity and Vitality in Elder breast cancer patients.

#### Impact of prediagnosis dietary intake on outcomes

The Arizona Food Frequency Questionnaire^33-35^ (FFQ) was selected by TEAL to characterize habitual dietary patterns for the 6 months preceding cancer diagnosis and nutrition intervention. This time period was chosen in an attempt to capture dietary intake prior to changes resulting from nutrition impact symptoms such as bloating, abdominal pain, and changes in bowel habits—symptoms often experienced prior to a diagnosis of ovarian cancer.^24^ FFQs assess retrospective dietary intake and describe general dietary patterns, have a relatively low participant burden, and are cost-effective. For comprehensive data on micro- and macronutrient intake, FFQs require matching reported quantities of foods and beverages to a relevant nutrient composition database. Analysis requires specialized software and often involves a trained data analyst.

#### Dietary intake pre- and postintervention

Because nutrition impact symptoms experienced during chemotherapy may cause participants to alter their usual dietary patterns,^28^ the working group recognized data collected through screeners or FFQs alone would not capture adequate detail to address this topic. To provide more time-sensitive and symptom-influenced intake data, 24-hour recalls are collected at baseline (presymptoms) and at the end of therapy (probable timepoint with highest symptom burden given compounding toxicities over treatment course). To balance quality dietary intake data with modest participant burden, all trials are collecting detailed short-term dietary intake data using at least two 24-hour food recalls at the beginning and at the end of the interventions using common or harmonizable instruments. Food recalls provide more detailed short-term intake than FFQs, not only capturing change in intake in relation to nutrition symptom burden but also supporting evaluation of diet quality and specific nutrients that may influence study outcomes.^36^

Completion of 24-hour food recalls requires participants to retrospectively report the time, type, amount, and preparation of foods, beverages, and supplements they consumed in the past 24 hours on random days over a specified period. Interviewers use a multipass method to ensure that all food and beverages are recorded. Contrary to 24-hour food recalls, food records are completed in real time, requiring participants to actively record their intake as it is consumed on specified days. Data collection using food records minimizes recall bias that may be present in retrospective food recalls. However, participants are aware of when the record will be collected, which may change actual dietary intake compared with unannounced food recalls.^36^ Similar to FFQs, 24-hour recalls and food records require use of an underlying nutrient database and specialized data analysis.

TEAL and THRIVE-65, which have nutrition-related intervention components, are conducting 24-hour recalls using the interviewer-assisted University of Arizona Behavioral Measurement and Interventions Shared Resource.^34^ Dietary recalls are completed with each participant on 2 randomly assigned, nonconsecutive days (1 weekday and 1 weekend day) at each measurement timepoint to capture dietary variety.^37^

The other 2 ENICTO trials that intervene only on exercise (ACTION and TNT) are collecting 24-hour dietary recalls using the online, free Automated Self-Administered 24-hour (ASA24) Dietary Assessment Tool (version 2022), developed by the NCI (Bethesda, MD),^38^ which is available in English, Spanish, and French.^38^ The working group recommended several ASA24 optional study parameters: intake time frames (midnight to midnight), unscheduled recall days, and the use of location and sleep modules, which were included by ACTION and TNT. The ASA24 recalls can be completed independently by participants through a web portal or with interviewer assistance (in-person or by telephone). Participants may complete the ASA24 as a food recall or as a food record, where foods and beverages are recorded in real time (ACTION) or entered post hoc into the ASA24 system by a trained interviewer or trial staff (TNT).

#### Eligibility for and adherence to intervention

Because THRIVE-65 and TEAL include nutrition-related components in their interventions, assessments to determine eligibility and adherence are included in characterization of adherence to the nutrition-related interventions and changes in diet quality between baseline and postchemotherapy timepoints. The THRIVE-65 nutrition-related intervention focuses solely on maintaining adequate dietary protein intake to support lean body mass and muscle gain (or maintenance) during the exercise intervention. Adherence to dietary protein intake recommendations is measured using an adaptation of a validated protein foods checklist.^39^ Participants in THRIVE-65 who do not eat at least 1.2 grams of protein per kilogram of body weight per day on 80% or more of assessed days receive dietary counseling over the telephone and a protein supplement. The same protein checklist is then repurposed to record protein food intake for measuring adherence to the intervention.

The TEAL dietary intervention addresses overall diet quality and has 5 individual dietary goals: to consume (1) at least 5 servings of vegetables and fruits per day; (2) at least 25 grams of fiber per day; (3) at least 1.2 grams of protein per kilogram body weight; (4) no more than 30 grams of added sugar per day; and (5) no more than 18 ounces of red and processed meats per week. TEAL utilizes a study goal-specific daily food journal that corresponds to the intervention goals. These food journals assess dietary components aligned with the TEAL intervention goals and will be used to calculate adherence to the nutrition-related component of the intervention.

#### Treatment-related nutrition impact symptoms

Nutrition impact symptoms are any symptoms that could potentially affect dietary intake, including nausea, changes in appetite, dry mouth, taste changes, difficulty swallowing, vomiting, mouth and/or throat sores, heartburn, diarrhea, constipation, and abdominal pain.

The DMWG collaborated with the Patient-Reported Measures Working Group to establish methods for data collection on nutrition impact symptoms during chemotherapy to understand variations in dietary intake during each treatment cycle. All 4 ENICTO trials use the self-report Patient-Reported Outcomes Common Terminology Criteria for Adverse Events (PRO-CTCAE),^40^ which was developed by the NCI as a free companion form to the widely used clinician-reported CTCAE to assess chemotoxicity symptoms. The PRO-CTCAE allows for systematic, yet adaptable, grading of toxicities by frequency and/or severity.^40^ Some fields also ask patients to report the degree of interference the symptoms present to their daily activities. All parameters can be graded using a validated composite-graded algorithm to classify individual symptoms as none, mild, moderate, or severe.^41^

It is important to note that the PRO-CTCAE asks respondents to identify symptoms experienced during the previous 7 days, prompting careful consideration of assessment timing. As chemotherapy-related side effects and/or toxicities are typically most severe in the first week postinfusion, asking participants to complete the PRO-CTCAE on day 1 of each chemotherapy cycle (prior to or during chemotherapy infusion) could result in underreporting symptoms. All studies are collecting PRO-CTCAE on day 8 of the first and last cycles of treatment at minimum or weekly to capture the period when patients are most likely to experience acute chemotherapy toxicity.

#### Risk or presence of malnutrition

As outlined in Figure 1, malnutrition is associated with greater treatment-related toxicity and decreased likelihood of completing anticancer treatment as initially prescribed.^42^ To assess risk or presence of malnutrition, 3 studies (TEAL, ACTION, and TNT) are collecting the patient-reported variables from the validated Patient-Generated Subjective Global Assessment at the baseline and postintervention assessments.^43^^,^^44^ This assessment was created for use in oncology research and clinical practice^44^ and includes patient-reported measures of weight history, food intake, symptoms, and activities and physical function.

#### Food security

The financial burden of cancer treatment (eg, cost of treatment, missing work, travel costs), known as financial toxicity, may impact patients’ food security, including the ability to purchase and access food.^45^^,^^46^ ACTION is using the US Department of Agriculture Economic Research Service 6-Item Short Form (USDA-6),^47^ which uses a subset of 6 questions from the larger 18-question household food security screener. The USDA-6 instrument focuses primarily on changes and frequency of changes in food intake, such as decreasing the size of meals because of food insecurity.^47^ The other 3 trials (THRIVE-65, TEAL, and TNT) are using the 2-item Hunger Vital Sign,^48^ which uses 2 questions from the larger USDA food security screener. The Hunger Vital Sign specifically asks whether participants worry about food running out and whether food actually ran out before they could afford to buy more.^48^ As food security was not a primary outcome of the overall studies, investigators chose validated instruments that minimized participant burden.

### Cross-consortium research

Common nutrition data elements within the consortium, as outlined in Table 2, will be pooled to create a deidentified, harmonized dataset for cross-consortium analyses addressing the DMWG priority topics. Data on dietary intake (macro- and micronutrients and food groups), supplement use, meal timing, and dietary patterns will be used to characterize dietary patterns and trends at baseline and throughout chemotherapy in all trials. Adherence to nutrition-related interventions in TEAL and THRIVE-65 can be calculated to evaluate associations between the intervention and treatment relative dose intensity. PRO-CTCAE data from all ENICTO trials will be used in analyses to determine the prevalence and longitudinal development of treatment-related nutrition impact symptoms. Data on the presence of malnutrition and/or food security will be used to analyze longitudinal changes in these domains associated with treatment in all the studies.

To ensure rigor in scientific reporting, all proposed cross-consortium analyses undergo a rigorous internal peer-review process by the Publications and Presentations committee (an interdisciplinary group of researchers, epidemiologists, and biostatisticians) prior to data analyses. All analyses resulting from cross-consortium nutrition data collection will address secondary research questions using observational data and will thus adhere to the Strengthening the Reporting of Observational Studies in Epidemiology statement^49^ to ensure transparency as recommended in the P2P report.^4^

### Advantages and challenges associated with standardization

The instruments described above were selected through a process that balanced the need for cross-consortium standardization with individual study design and the need to minimize participant burden for participants undergoing active cancer treatment. Although many common data elements and standard operating procedures were kept consistent, there are notable advantages and challenges associated with the instruments.

The current approach adopted by the ENICTO Consortium to address knowledge gaps in nutrition during chemotherapy has several strengths. First, if the individual studies meet their recruitment goals, the harmonized dataset will have nutrition-related data on nearly 1000 study participants receiving chemotherapy. The collection of common nutrition data elements, as described in Table 2, will allow for pooling of data across trials thereby increasing statistical power and facilitating the ability to conduct stratified analyses across different subgroups. Second, the inclusion of longitudinal, patient-reported nutrition impact symptoms through PRO-CTCAE across chemotherapy regimens offers a valuable opportunity to track changes in symptoms relevant to future interventions and clinical practice. Furthermore, the diversity in cancer types, trial locations across the United States, and patient populations is a unique feature of the consortium that will enhance the generalizability of the findings. Finally, collection of detailed prospective dietary and clinical data together is available in few other trials conducted during active treatment. Together, the unique strengths of the ENICTO Consortium will allow for the interrogation of relationships between diet and treatment outcomes presented in the conceptual framework (Figure 1).

A primary challenge for the ENICTO Consortium studies was to balance the participant burden and time constraints of dietary intake assessment with other demands of the studies and the need for more data to guide future nutrition interventions. Repeated, detailed dietary intake assessment would result in unacceptable participant burden in some studies. This concern limited collection of 24-hour recalls to 2 days each at the pre-and postintervention timepoints. Preintervention habitual dietary intake data could not be collected by all studies, precluding cross-consortium analysis of dietary exposure during this period. Finally, dietary intake occurring during the chemotherapy cycles was identified as a priority; however, repeated dietary assessment during chemotherapy was determined not to be feasible for all studies. Smaller challenges include differences in food composition databases and data collection methods. We will conduct and report the findings of sensitivity analyses across the ENICTO studies to determine whether the method of dietary intake reporting impacts study findings.

## Conclusion and future directions

The ENICTO Consortium represents a pivotal national endeavor to bring together investigators of 4 randomized controlled trials and address the prevailing limitations in the field of oncology nutrition. Leveraging the collective expertise of the DMWG, we have outlined research priorities, standardized dietary assessment methods, and identified key outcomes of interest across all studies within the consortium. At the conclusion of the ENICTO Consortium funding period (approximately July 2027), a harmonized, deidentified dataset will be made available to the broader scientific community through one of the controlled-access data repositories maintained by the National Institutes of Health. This resource will facilitate the exploration of additional research questions and catalyze further progress in the field of nutrition oncology. We call on fellow oncology nutrition researchers in the oncology lifestyle intervention field to adopt approaches similar to those outlined here, building off a clear conceptual framework and prioritizing data harmonization efforts.

## Supplementary Material

djaf143_Supplementary_Data

## Data Availability

In accordance with the NIH Public Access Policy, we will provide all manuscripts to PubMed Central including this manuscript. At the end of the funding period, a de-identified dataset will be made available to the broader research community through one of the controlled-access data repositories maintained by the NIH, such as the database of Genotypes and Phenotypes (dbGAP).

## References

[1] Hiatt RA , Clayton MF, Collins KK, et al The Pathways to Prevention program: nutrition as prevention for improved cancer outcomes. JNCI J Natl Cancer Inst. 2023;115:886-895. 37212639 10.1093/jnci/djad079 PMC10407697

[2] Ligibel JA , Bohlke K, May AM, et al Exercise, diet, and weight management during cancer treatment: ASCO guideline. J Clin Oncol. 2022;40:2491-2507. 35576506 10.1200/JCO.22.00687

[3] Rock CL , Thomson CA, Sullivan KR, et al American Cancer Society nutrition and physical activity guideline for cancer survivors. CA Cancer J Clin. 2022;72:230-262. 35294043 10.3322/caac.21719

[4] Parsons HM , Forte ML, Abdi HI, et al Nutrition as prevention for improved cancer health outcomes: a systematic literature review. JNCI Cancer Spectr. 2023;7:pkad035. 10.1093/jncics/pkad035 PMC10290234 37212631

[5] Schmitz KH , Brown JC, Irwin ML, et al Exercise and nutrition to improve cancer treatment-related outcomes (ENICTO). J Natl Cancer Inst. 2025;117:9-19. 39118255 10.1093/jnci/djae177 PMC11717426

[6] Nielson CM , Bylsma LC, Fryzek JP, et al Relative dose intensity of chemotherapy and survival in patients with advanced stage solid tumor cancer: a systematic review and meta-analysis. Oncologist. 2021;26:e1609-e1618. 33973301 10.1002/onco.13822 PMC8417866

[7] Weycker D , Barron R, Edelsberg J, et al Incidence of reduced chemotherapy relative dose intensity among women with early stage breast cancer in US clinical practice. Breast Cancer Res Treat. 2012;133:301-310. 22270932 10.1007/s10549-011-1949-5

[8] Purcell SA , Kok DE, Ketterl T, et al Pharmacokinetics of cancer therapeutics and energy balance: the role of diet intake, energy expenditure, and body composition. J Natl Cancer Inst Monogr. 2023;2023:3-11. 37139976 10.1093/jncimonographs/lgad010 PMC10157766

[9] Cespedes Feliciano EM , Chen WY, Lee V, et al Body composition, adherence to anthracycline and taxane-based chemotherapy, and survival after nonmetastatic breast cancer. JAMA Oncol. 2020;6:264-270. 31804676 10.1001/jamaoncol.2019.4668 PMC6902178

[10] Prado CM , Baracos VE, McCargar LJ, et al Sarcopenia as a determinant of chemotherapy toxicity and time to tumor progression in metastatic breast cancer patients receiving capecitabine treatment. Clin Cancer Res. 2009;15:2920-2926. 19351764 10.1158/1078-0432.CCR-08-2242

[11] Guyton AC , Hall JE. Textbook of Medical Physiology. 11th ed. Elsevier Saunders; 2006.

[12] Beasley JM , Shikany JM, Thomson CA. The role of dietary protein intake in the prevention of sarcopenia of aging. Nutr Clin Pract. 2013;28:684-690. 24163319 10.1177/0884533613507607 PMC3928027

[13] Surov A , Pech M, Gessner D, et al Low skeletal muscle mass is a predictor of treatment related toxicity in oncologic patients. A meta-analysis. Clin Nutr. 2021;40:5298-5310. 34536638 10.1016/j.clnu.2021.08.023

[14] Williams GR , Al-Obaidi M, Rower J, et al *Does Oxaliplatin Pharmacokinetics (PKs) Explain Associations between Body Composition and Chemotherapy Toxicity Risk in Older Adults with Gastrointestinal (GI) Cancers?* Wolters Kluwer Health; 2021.

[15] Camaschella C. Iron-deficiency anemia. New Engl J Med. 2015;372:1832-1843. 25946282 10.1056/NEJMra1401038

[16] Rodgers GM 3rd , Becker PS, Blinder M, et al Cancer- and chemotherapy-induced anemia. J Natl Compr Canc Netw. 2012;10:628-653. 22570293 10.6004/jnccn.2012.0064

[17] Heilfort L , Kutschan S, Dörfler J, et al A systematic review of the benefit of B-vitamins as a complementary treatment in cancer patients. Nutr Cancer. 2023;75:33-47. 35819060 10.1080/01635581.2022.2098348

[18] Zhou L , Yang H, Wang J, et al The therapeutic potential of antioxidants in chemotherapy-induced peripheral neuropathy: evidence from preclinical and clinical studies. Neurotherapeutics. 2023;20:339-358. 36735180 10.1007/s13311-023-01346-8 PMC10121987

[19] Zhu W , Xu B. Association of pretreatment anemia with pathological response and survival of breast cancer patients treated with neoadjuvant chemotherapy: a population-based study. PLoS One. 2015;10:e0136268. 26291454 10.1371/journal.pone.0136268 PMC4546363

[20] Chaumard N , Limat S, Villanueva C, et al Incidence and risk factors of anemia in patients with early breast cancer treated by adjuvant chemotherapy. Breast. 2012;21:464-467. 22123411 10.1016/j.breast.2011.10.009

[21] Gianni L , Cole BF, Panzini I, et al Anemia during adjuvant non-taxane chemotherapy for early breast cancer: incidence and risk factors from two trials of the International Breast Cancer Study Group. Support Care Cancer. 2008;16:67-74. 17629752 10.1007/s00520-007-0295-y

[22] Hufnagel DH , Mehta ST, Ezekwe C, et al Prevalence of anemia and compliance with NCCN guidelines for evaluation and treatment of anemia in patients with gynecologic cancer. J Natl Compr Canc Netw. 2021;19:513-520. 33524941 10.6004/jnccn.2020.7638

[23] Sharma P , Georgy JT, Andrews AG, et al Anemia requiring transfusion in breast cancer patients on dose-dense chemotherapy: prevalence, risk factors, cost and effect on disease outcome. Support Care Cancer. 2022;30:5519-5526. 35314996 10.1007/s00520-022-06970-2 PMC8937495

[24] Ebell MH , Culp MB, Radke TJ. A systematic review of symptoms for the diagnosis of ovarian cancer. Am J Prev Med. 2016;50:384-394. 26541098 10.1016/j.amepre.2015.09.023

[25] Smith D , Ballal M, Hodder R, et al Symptomatic presentation of early colorectal cancer. Ann R Coll Surg Engl. 2006;88:185-190. 16551416 10.1308/003588406X94904 PMC1964069

[26] Fritz CDL , Otegbeye EE, Zong X, et al Red-flag signs and symptoms for earlier diagnosis of early-onset colorectal cancer. J Natl Cancer Inst. 2023;115:909-916. 37138415 10.1093/jnci/djad068 PMC10407716

[27] Arends J , Bachmann P, Baracos V, et al ESPEN guidelines on nutrition in cancer patients. Clin Nutr. 2017;36:11-48. 27637832 10.1016/j.clnu.2016.07.015

[28] Milliron BJ , Packel L, Dychtwald D, et al When eating becomes torturous: understanding nutrition-related cancer treatment side effects among individuals with cancer and their caregivers. Nutrients. 2022;14:356-376. 35057538 10.3390/nu14020356 PMC8781744

[29] Coa KI , Epstein JB, Ettinger D, et al The impact of cancer treatment on the diets and food preferences of patients receiving outpatient treatment. Nutr Cancer. 2015;67:339-353. 25664980 10.1080/01635581.2015.990577 PMC4353259

[30] Pereira AA , Reis ESD, Guilarducci MJ, et al Food aversion during cancer treatment: a systematic review. Nutr Cancer. 2023;75:389-401. 36382624 10.1080/01635581.2022.2129079

[31] McQuade RM , Stojanovska V, Abalo R, et al Chemotherapy-induced constipation and diarrhea: pathophysiology, current and emerging treatments. Front Pharmacol. 2016;7:414. 27857691 10.3389/fphar.2016.00414 PMC5093116

[32] Larkin PJ , Sykes NP, Centeno C, et al; European Consensus Group on Constipation in Palliative Care. The management of constipation in palliative care: clinical practice recommendations. Palliat Med. 2008;22:796-807. 18838491 10.1177/0269216308096908

[33] Martinez ME , Marshall JR, Graver E, et al Reliability and validity of a self-administered food frequency questionnaire in a chemoprevention trial of adenoma recurrence. Cancer Epidemiol Biomarkers Prev. 1999;8:941-946. 10548325

[34] University of Arizona Cancer Center. Behavioral measurement and interventions. Accessed April 1, 2024. https://cancercenter.arizona.edu/researchers/shared-resources/behavioral-measurement-and-interventions

[35] Thomson CA , Giuliano A, Rock CL, et al Measuring dietary change in a diet intervention trial: comparing food frequency questionnaire and dietary recalls. Am J Epidemiol. 2003;157:754-762. 12697580 10.1093/aje/kwg025

[36] Willett W. Nutritional Epidemiology. Oxford University Press; 2012.

[37] Jonnalagadda SS , Mitchell DC, Smiciklas-Wright H, et al Accuracy of energy intake data estimated by a multiplepass, 24-hour dietary recall technique. J Am Diet Assoc. 2000;100:303-311. 10719403 10.1016/s0002-8223(00)00095-x

[38] National Cancer Institute. Automated Self-Administered 24-Hour (ASA24^®^) dietary assessment tool. Accessed March 17, 2024. https://epi.grants.cancer.gov/asa24/

[39] Morin P , Herrmann F, Ammann P, et al A rapid self-administered food frequency questionnaire for the evaluation of dietary protein intake. Clin Nutr. 2005;24:768-774. 15908055 10.1016/j.clnu.2005.03.002

[40] National Cancer Institute. Division of Cancer Control and Population Sciences: PRO-CTCAE, data & tools. Accessed December 14, 2023. https://healthcaredelivery.cancer.gov/pro-ctcae/

[41] Basch E , Becker C, Rogak LJ, et al Composite grading algorithm for the National Cancer Institute’s Patient-Reported Outcomes version of the Common Terminology Criteria for Adverse Events (PRO-CTCAE). Clinical Trials. 2021;18:104-114. 33258687 10.1177/1740774520975120 PMC7878323

[42] Klute KA , Brouwer J, Jhawer M, et al Chemotherapy dose intensity predicted by baseline nutrition assessment in gastrointestinal malignancies: a multicentre analysis. Eur J Cancer. 2016;63:189-200. 27362999 10.1016/j.ejca.2016.05.011

[43] Jager-Wittenaar H , Ottery FD. Assessing nutritional status in cancer: role of the patient-generated subjective global assessment. Curr Opin Clin Nutr Metab Care. 2017;20:322-329. 28562490 10.1097/MCO.0000000000000389

[44] Ottery FD. Definition of standardized nutritional assessment and interventional pathways in oncology. Nutrition. 1996;12:S15-9. 8850213 10.1016/0899-9007(96)90011-8

[45] Khan HM , Ramsey S, Shankaran V. Financial toxicity in cancer care: implications for clinical care and potential practice solutions. J Clin Oncol. 2023;41:3051-3058. 37071839 10.1200/JCO.22.01799

[46] McDougall JA , Anderson J, Adler Jaffe S, et al Food insecurity and forgone medical care among cancer survivors. J Clin Oncol Oncol Pract. 2020;16:e922-e932. 10.1200/JOP.19.00736 PMC7489488 32384017

[47] Blumberg SJ , Bialostosky K, Hamilton WL, et al The effectiveness of a short form of the Household Food Security Scale. Am J Public Health. 1999;89:1231-1234. 10432912 10.2105/ajph.89.8.1231 PMC1508674

[48] Hager ER , Quigg AM, Black MM, et al Development and validity of a 2-item screen to identify families at risk for food insecurity. Pediatrics. 2010;126:e26-e32. 20595453 10.1542/peds.2009-3146

[49] von Elm E , Altman DG, Egger M, et al; STROBE Initiative The Strengthening the Reporting of Observational Studies in Epidemiology (STROBE) statement: guidelines for reporting observational studies. J Clin Epidemiol. 2008;61:344-349. 18313558 10.1016/j.jclinepi.2007.11.008

