# Supplementary material for: Addressing conceptual and design gaps in the oncology nutrition evidence base during chemotherapy: contributions of the Exercise and Nutrition Interventions to Improve Cancer Treatment-Related Outcomes Consortium
Source: J Natl Cancer Inst. 2025 Jul 18;117(12):2441–51. doi: 10.1093/jnci/djaf143 (PMC12682377; doi:10.1093/jnci/djaf143)
Supplement: djaf143_Supplementary_Data [file djaf143_supplementary_data.pdf]

Supplementary Materials

Supplementary Table 1. Description of ENICTO Study Interventions

Study	Population	Overall study design and exercise intervention	Nutrition Intervention
ACTION NCT05773144<br><br>Goal 219 participants	Colon<br><br>Age ≥ 18 years old<br><br>Histologically confirmed stage II or III colon cancer<br><br>Completed surgical resection with curative intent<br><br>Plan to initiate chemotherapy	Bayesian, multi-stage, response-adaptive randomized design<br><br>Attention control (progressive stretching) or aerobic exercise (treadmill delivered to the participant's home)<br><br>Aerobic exercise targets will follow a chemotherapy-periodized progression as follows:<br>(i) 75 mins/week,<br>(ii) 150 mins/week,<br>(iii) 225 mins/week,<br>(iv) 300 mins/week of aerobic exercise	No specific nutrition intervention
TEAL NCT05761561<br><br>Goal 200 participants	Ovarian<br><br>Diagnosis of epithelial ovarian cancer, fallopian tube or primary peritoneal carcinoma, stage I-IV<br><br>Scheduled to receive neoadjuvant/adjuvant chemotherapy	2-arm RCT<br>(*medical nutrition therapy and exercise intervention vs. usual care*)<br><br>18-21 weekly sessions with RD and exercise trainer for the duration of chemotherapy treatments<br><br>Exercise goals:<br>≥150 min moderate intensity physical activity per week<br>2 times/week strength training<br>Reduce sedentary time	Nutrition goals:<br>≥ 5 servings of vegetable/fruit per day<br>≥ 25 grams fiber/day<br>≥1.2 grams/kilogram per day protein<br>≤30 grams of added sugar per day<br>≤18 ounces of red and processed meats per week

THRIVE-65<br>NCT05535192<br><br>Goal 270 participants	Female breast<br><br>Age ≥65 years<br><br>Stage I-III invasive breast cancer<br><br>BMI 18 – 50 kg/m^2<br><br>Scheduled to receive a cytotoxic chemotherapy regimen of at least 10 weeks duration	2-arm RCT<br>(*exercise intervention vs. health education and support control group*)<br><br>Progressive resistance exercise, moderate-intensity aerobic exercise (30 minutes, 3 times/week)<br><br>One to two in-person exercise sessions, followed by 2 times/week home-based telehealth supervised exercise sessions and remotely delivered dietary assessment and recommendations.	Protein intake support 1.2 grams/kilogram per day based on protein foods checklist
TNT<br>NCT05789485<br><br>Goal 216 participants	Gastrointestinal<br><br>Age ≥ 18 years old<br><br>Locally advanced gastrointestinal cancer initiating total neoadjuvant therapy	3-arm RCT<br><br>(i) 90 mins/week,<br>(ii) 150 mins/week, or<br>(iii) 300 mins/week<br>of individualized, structured aerobic training (treadmill walking)	No specific nutrition intervention

Adaptive Randomization of Aerobic Exercise During Chemotherapy in Colon Cancer (ACTION); Trial of Exercise And Lifestyle (TEAL); TeleHealth Resistance exercise Intervention to preserve dose intensity and Vitality in Elder breast cancer patients (THRIVE-65); Tele-exercise during Neoadjuvant chemotherapy Trial (TNT);

ENICTO Research Group

Kaiser Permamente
(Oakland, CA) – ACTION
Bette Caan, DrPH*
Sydney Anderson, MS, OTR/L
Harmenjit Bahia, BA
Adrienne Castillo, MS, RD
Elizabeth Feliciano, ScD, MS
Kristina Johnson, MPH
Michelle Ross, MPH
Erin Weltzein, BA

Pennington Biomedical Research Center
(Baton Rouge, LA) - ACTION
Justin C. Brown, PhD*
Baylea Albarado, BS
Stephanie Compton, PhD, RD, LDN
Tamara Green, BS
Ryan Nash, MS
Phillip Nauta, MBA
Meredith Welch, BSN, RN
Shengping Yang, PhD

Dana-Farber
(Boston, MA) – ACTION
Jeffrey A. Meyerhardt, MD*
Christina M. Dieli-Conwright, PhD
Danny Nguyen, BS
Amalia Perez Pena, BS

Lousiana State University
(Baton Rouge, LA) - ACTION
Guillaume Spielmann, PhD
Youyoung Kim, MS

UC Berkeley
(Berkeley, CA) - TEAL
William J. Evans, PhD

University of Arizona
(Tucson, AZ) - TEAL
Jennifer W. Bea, PhD
Robert M. Blew, MS
Cynthia A. Thomson, PhD, RDN

University of Miami
(Miami, FL) - TEAL
Tracy E. Crane, PhD, RDN*
Atif Bhatti, BS
Reanna Clavon, MPH
Stefan Spee Erlandsen, MSEd
Sarah Freylersythe, BS, NBC-HWC
Kenna Hollander, BS
Melissa Lopez-Pentecost, PhD, RDN
Frank J. Penedo, PhD
LaShae Rolle, MPH, CPH
Paola Rossi, MD
Matthew Schlumbrecht, MD
Madalyn Wheeler, MS

Yale University
(New Haven, CT) – TEAL
Melinda L. Irwin, PhD, MPH*
Anlan Cao, MBBS
Brenda Cartmel, PhD
Leah M. Ferrucci, PhD, MPH
Linda Gottlieb, MA, CPT, CET
Maura Harrigan, MS, RDN, CSO
Fang-Yong Li, MS, MPH
Courtney McGowan, MS, RDN, CSO, CNSC
Leah Puklin, MPH
Elena Ratner, MD, MBA
Tara Sanft, MD
Michelle Zupa, BS

Case Western
(Cleveland, OH) – THRIVE-65
Nathan A. Berger, MD*
Stephen Cerne, B.S., ACSM/ACS-CET, NSCA-CPT
Carissa Mills, BSN
Sandy Conochan, ATC, PTA
Jasmin Hundal, MD
Cynthia Owusu, MD, MS
John Pink, PhD

Dana-Farber
(Boston, MA) – THRIVE-65
Jennifer A. Ligibel, MD*
Nancy Campbell, MS
Kaedryn DiGuglielmo, MPH
Wendy Kemp, BS
Christopher Maples-Campbell, MS
Truong Nguyen, BS
Jay Oppenheim, MS
Anna Tanasijevic, MPH

* denotes Multiple Principal Investigator

ENICTO Research Group

University of Arizona
(Tucson, AZ) – THRIVE-65
Cynthia Thomson, PhD, RDN
Angela Yung, MBA

MD Anderson Cancer Center
(Houston, TX) – THRIVE-65
Karen Basen-Engquist, PhD
Preena Loomba, PhD

Penn State University
(State College, PA) – THRIVE-65
Vernon M. Chinchilli, PhD

University of Pittsburgh
(Pittsburgh, PA) – THRIVE-65
Kathryn H. Schmitz, PhD*
Jenna Binder, PhD
Shawna E. Doerksen, PhD
Julia Foldi, MD
Sara Garrett, PhD
Raymond Scalise, MS
Michele Sobolewski, MS
Lacey White, MS

Memorial Sloan Kettering
(New York, NY) - TNT
Jessica M. Scott, PhD*
Andrea Cercek, PhD*
Sheng F. Cai, MD, PhD
Stephanie Cao, BA
Helena Furberg, PhD
Jenna Harrison, BS
Lee W. Jones, PhD
Catherine Lee, BS
Ross Levine, MD
Meghan Michalski, MA
Chaya S. Moskowitz, PhD
Robert Novo, BS
Julia Rabazzi, BS
Kurtis Stoeckel, BS
Talya Salz, PhD
Martin R. Weiser, MD
Anthony F. Yu, MD

University of Alabama at Birmingham
(Birmingham, AL) - TNT
Wendy Demark-Wahnefried, PhD, RD

George Washington University
(Washington, DC) – Coordinating Center
Kim Robien, PhD, RD, CSO*
Scott R. Evans, PhD*
Loretta DiPietro, PhD
Bao Duong, BS
Sharon L. Edelstein, ScM
Lorens Helmchen, PhD
Daisy Le, PhD
Caitlin McCleary, MPH
Ashley H. Tjaden, MPH
Heather Wopat, MSN

University of California San Diego
(La Jolla, CA) – Coordinating Center
Borsika A. Rabin, PhD, PharmD

National Cancer Institute
(Bethesda, MD)
Frank M. Perna, EdD, PhD
Tanya Agurs-Collins, RD, PhD
Susan M. Czajkowski, PhD
Joanne Elena, PhD, MPH
Linda C. Nebeling, PhD, RD, FAND
Wynne E. Norton, PhD

* Denotes Multiple Principal Investigator
